# “Prison life is very hard and it’s made harder if you’re isolated”: COVID-19 risk mitigation strategies and the mental health of incarcerated women in California

**DOI:** 10.1108/IJPH-09-2021-0093

**Published:** 2022-11-21

**Authors:** Jennifer E. James, Leslie Riddle, Giselle Perez-Aguilar

**Affiliations:** Institute for Health and Aging, University of California, San Francisco, California, USA.; Department of Humanities and Social Sciences, University of California, San Francisco, California, USA.; Department of Social and Behavioral Sciences, University of California, San Francisco, California, USA.

**Keywords:** Mental health, Qualitative research, Women prisoners, Correctional health care, Elderly prisoners, COVID-19

## Abstract

**Purpose –:**

This study aims to describe the COVID-19 risk mitigation strategies implemented in California prisons and the impact of these policies on the mental health of incarcerated women.

**Design/methodology/approach –:**

The authors conducted semi-structured qualitative interviews with ten women who were over the age of 50 and/or had a chronic illness and had been incarcerated in California prisons during the COVID-19 pandemic. The authors also interviewed ten health-care providers working in California jails or prisons during the pandemic. Interviews were analyzed using a grounded theory coding framework and triangulated with fieldnotes from ethnographic observations of medical and legal advocacy efforts during the pandemic.

**Findings –:**

Participants described being locked in their cells for 23 hours per day or more, often for days, weeks or even months at a time in an effort to reduce the spread of COVID-19. For many participants, these lockdowns and the resulting isolation from loved ones both inside and outside of the prison were detrimental to both their physical and mental health. Participants reported that access to mental health care for those in the general population was limited prior to the pandemic, and that COVID-19 risk mitigation strategies, including the cessation of group programs and shift to cell-front mental health services, created further barriers.

**Originality/value –:**

There has been little qualitative research on the mental health effects of the COVID-19 pandemic on incarcerated populations. This paper provides insight into the mental health effects of both the COVID-19 pandemic and COVID-19 risk mitigation strategies for the structurally vulnerable older women incarcerated in California prisons.

## Introduction

The COVID-19 pandemic has wreaked havoc on structurally vulnerable populations in the USA, with a disproportionate impact on Black, Indigenous and People of Color (BIPOC) (Laster Pirtle, 2020). Nowhere can this be seen more clearly than in correctional facilities, where COVID-19 has spread with abandon. J. Clark Kelso, the Federal Receiver overseeing health care across the California Department of Corrections and Rehabilitation (CDCR), described the catastrophic potential of the pandemic noting, “If the coronavirus were designing its ideal home, it would build a prison” (*Coleman v Newsom*, 2021). Indeed, early in the pandemic, health experts raised alarm about the unique risks the virus would undoubtedly pose for incarcerated populations (Hewson *et al*., 2020; McCoy *et al*., 2020; Stewart *et al*., 2020), given that the structural realities of carceral settings, such as crowded conditions, limited sanitation, frequent turnover and a high prevalence of chronic medical conditions (Binswanger *et al*., 2012), were thought to increase outbreak likelihood and severity. Those prescient warnings were not acted upon. In 2020, prisons and jails accounted for 85 of the top 100 COVID-19 clusters in the USA, and seven out of the ten largest prison outbreaks occurred in California (Times, 2020). As of February 17, 2022, there have been 71,372 cases of COVID-19 in California State Prisons and 249 deaths (Population COVID-19 Tracking, 2022).

There has been limited research to date on the mental health impact of COVID-19 on those who are incarcerated; yet, the implications of the pandemic for incarcerated populations are clear. Incarceration is a stressful experience even in normal times (Massoglia and Remster, 2019), and chronic activation of the stress response has been associated with poor physical and mental health outcomes (Porter, 2019). High baseline rates of mental illness among incarcerated populations have also been well documented, with an estimated one in seven incarcerated people suffering from major depression or psychosis (Fazel *et al*., 2016). From the limited research published on the mental health toll of COVID-19 for incarcerated populations, several key challenges have been identified, including social distancing, isolation, cessation of prison visits and reduced or discontinued mental health services (Johnson *et al*., 2021). Indeed, COVID-19 risk mitigation strategies in carceral settings have largely relied on the same practices recommended for non-congregate living spaces such as social distancing (World Health Organization, 2020), despite stark differences in these environments. Studies on the longer-term effects of COVID-19 on the mental health of older adults specifically, who comprise a growing proportion of the incarcerated population (Prost *et al*., 2021), are also limited, despite acknowledgment that many older adults do not have adequate material, social, physical or cognitive resources to deal with the stress of the pandemic (Vahia *et al*., 2020). Research has shown that, compared with their younger counterparts, mental health conditions are already common for older incarcerated adults, especially anxiety and depression (Lane *et al*., 2020; Skarupski *et al*., 2018).

### Delivery of mental health services in California prisons

According to a 2017 Stanford Justice Advocacy Project analysis of CDCR data, within the past ten years, the California State Prison population has dropped, while the number of incarcerated individuals with mental health issues has risen; as of 2017, the number of incarcerated people with serious mental illnesses reached about 32% of the prison population in California (2017). For individuals incarcerated in CDCR prisons, mental health treatment is administered through a referral process categorized as Emergent, Urgent or Routine (Mehta *et al*., 2021). Prior to the pandemic, most individuals with lower-level mental health-care needs received treatment via group programming. In the early days of the pandemic, most group programs ceased system-wide, and have yet to be fully restored across all institutions, due to current outbreak status (Mehta *et al*., 2021). CDCR provided packet-based in-cell activities in an effort to mitigate the loss of groups. Across the system, transfers between facilities were restricted except for emergency situations, which left hundreds of individuals incarcerated at facilities unable to have their mental health-care needs met. Additionally, there was a major shift from out-of-cell mental health services to cell-front care, which corresponded with plummeting referrals for inpatient services. These new procedures effectively limited mental health services unless clinically urgent (Mehta *et al*., 2021). CDCR notes that their mental health staffing, like that in many non-carceral health settings, was adversely impacted during the pandemic due to staff infections, mandatory quarantine periods and providers avoiding prison facilities due to their own health concerns. CDCR implemented a telepsychiatry program in 2013, which was expanded in May of 2020, but, as of 2021, was still not available at all institutions (Mehta *et al*., 2021).

The reliance on lockdowns, quarantines and medical isolation as critical COVID-19 risk mitigation strategies in prisons, combined with mental health staffing shortages and the abrupt shift away from group therapy during the pandemic has led to concerns about the mental health implications of the pandemic on the incarcerated population. The negative impact of social isolation (Freak-Poli *et al*., 2021; World Health Organization, 2015), with limited access to enrichment programs, exercise, staff or contact with other incarcerated people (Metzner and Dvoskin, 2006), has been well established (Dellazizzo *et al*., 2020; Haney, 2018; Luigi *et al*., 2020; Reiter *et al*., 2020). The unique implications of COVID-19-related isolation for older incarcerated women has yet to be explored qualitatively. In this paper, we describe the mental health effects of the COVID-19 pandemic and COVID-19 risk mitigation strategies on older women with chronic illnesses incarcerated in California State Prisons.

## Methods

The findings described here stem from a larger research project on the health of currently and formerly incarcerated older adults during the COVID-19 pandemic, which included 18 months of ethnographic observations of legal and medical advocacy work, focus groups with organizers and activists and interviews with key informants. For this paper, we present findings from interviews conducted with ten women who were incarcerated in five different California State Prisons and one ICE detention facility during the first year of the COVID-19 pandemic and ten health-care providers working in six prisons and one jail during this time [1] (Table 1). The sample size was largely driven by sampling strategy and the availability of the respondents. We aimed to recruit participants who were released from prison across the first year of the pandemic (2020) and health-care providers who worked across several disciplines and institutions. The small sample size is appropriate, given the nature of this exploratory, qualitative research and our aim of exploring each participant’s narrative in depth and enabling significant reflection, dialogue and time on each transcript.

We recruited participants via snowball sampling with the help of community organizations and partners and direct outreach to those in our personal and professional networks. Semi-structured interviews were conducted via Zoom or phone and lasted 1 to 1.5 hours. Interviews were audio-recorded and transcribed verbatim, then analyzed using techniques based in grounded theory (Corbin and Strauss, 2008). Three researchers collaborated on data analysis for this project. To start, the authors read two to three transcripts independently and engaged in a process of open coding (Bryant and Charmaz, 2007) to create an initial set of inductive codes grounded in the data and deductive codes based on prior research with similar populations. We discussed our individual lists of codes and worked collaboratively to develop a code list that was continually adapted over the course of our analysis. We then engaged in independent parallel coding of each transcript using the qualitative analysis software Atlas.ti. We met regularly as a team to discuss any discrepancies in coding and come to consensus on codes and definitions. Reports were generated for each code, and the quotations associated with each code were then further refined into categories and then into themes in an iterative process. These themes were discussed in-depth in research meetings. Our team included diversity of race, ethnicity, discipline and experience with research, allowing us to approach the data from many perspectives.

This study was approved by the University of California, San Francisco, IRB.

## Findings

### Fear and uncertainty: “How are we going to keep ourselves safe?”

The novel nature of COVID-19 has presented a global challenge as information and official guidance has evolved and, often, been contradicted. Many incarcerated people were watching the news and hearing about COVID-19, but with even less access to information than those living in the community. As one 42-year-old woman, Beyond Blessed, described:

When COVID hit, we were watching it in the news and all the officers were barely […] They weren’t giving us any information about what the virus is about, what measures [they] are taking to prevent us from getting sick. They weren’t very open about it. And I believe it’s because they weren’t briefed and they [didn’t] know. But some of them were rude [and would] say, “hell if I know”, or “I don’t know, stop asking me.”

In her estimation, the prison did not offer enough information – though presumably correctional officers may have also had little information in the early days of the pandemic. This lack of information only increased fear and anxiety. As Shay Shay, 48, described:

I remember we was watching TV and they had a breaking news about this virus and they said it’s hitting California. So at first start we were scared because we didn’t know if it got into prison, what would happen to us. We didn’t know if the COs were bringing in on their own. We didn’t know anything. We just knew we had to keep ourselves protected.

This reflection echoed sentiments from other formerly incarcerated women who felt like there was not enough information offered, and that it was up to them to figure out how to keep themselves safe; they could not depend on the staff or institution to protect them.

Cece, 46, became ill with what she believed to be COVID-19 in January 2020, before the pandemic was on anyone’s radar. She described the fear and uncertainty she felt about what was happening and the severity of her symptoms:

They didn’t even know what it was. They were saying everybody had a flu in the yard and people were dying […] I can honestly say, that was probably the worst pain [and] I’ve been shot three times […] I was scared of it because I didn’t know what it was.

Even as more information about COVID-19 came down through official channels and policies began to be implemented to reduce the spread of the virus, concerns about incarcerated women’s safety persisted. Indeed, participants, both those who were incarcerated and health-care providers working in the facilities, cited the failure of CDCR to enforce these policies As Mariella explained:

The staff wouldn’t wear [masks] […] My biggest issue with CDCR is that they didn’t demand that their employees be responsible and follow the restrictions that were put in place because you would hear staff talk about, for months, “oh, I just went to see my brother”. You don’t live with your brother, what are you going to see them for, I’m thinking in my head. But they don’t care because it’s not their family that they are infecting. It’s us.

Several providers also referenced the refusal of correctional staff to wear masks. As one nurse reflected, “corrections officers were not inclined to believe that COVID was contagious, I guess. So it was just interesting, people’s comfort levels with not wearing masks there.” Similarly, one physician said, “I would hear from the providers who were like wearing full plastic head to toe […] they said the staff, the officers, weren’t always wearing masks.” This physician described a culture of COVID-19 denial at two different prisons where she worked during the pandemic, and being retaliated against for reporting staff who refused to wear masks.

Providers also described the lack of clear policies in the first year of the COVID-19 pandemic. One physician noted feeling like the institution was “completely abandoned.” She went on to say, “I felt like all the inmates were trapped in their cells to get COVID […] I remember being horrified by the lack of fear that the executives had.” A nurse who began her work in the prison seven months into the pandemic noted, “it was really shocking like how terrible COVID had been in that facility and how their management of it just felt like a mess.” The lack of information and inconsistent enforcement of policy trickled down to the people who were incarcerated. Beyond Blessed described the chaos of the early days of the pandemic:

They are stressed out, they’re scared, they don’t know what’s going on with the world. They are worried about their family. Everybody’s running out of water, toilet paper. People were hoarding it. They had to search the rooms. There was just a lot of crazy stuff going on.

Unable to seek out additional information from the internet or other trusted sources, many incarcerated women felt an increasing sense of panic.

### Lockdowns: “we were stuck in a room and we had to stay locked in all the time. And for some people, that’s really hard”

As described above, a crucial strategy of COVID-19 risk mitigation inside prisons and jails were lockdowns; incarcerated people would be kept in their cells for days, weeks or even months at a time to reduce contact between individuals and spread of the virus if someone were to become infected. Content, 59, recalled the first lockdown of the pandemic. An individual tested positive in one of the factories; given that workers in the factory came from across the prison, there was risk of exposure in nearly every unit. Rumors began to spread that something might be happening in response to the potential outbreak. She described:

And so the executive WAC [Women’s Advisory Council] kind of gave us a heads up, like “hey you guys, you better get everything in order because they’re getting ready to slam everybody down”. And at that point I was hustling trying to take my shower and, sure enough, when they locked us in for our 4 o’clock count, we were locked in.

Lockdowns are not a unique phenomenon of the pandemic; most incarcerated people have experienced them due to other contagious illnesses, weather or security concerns. The people we interviewed were clear that the prospect of extended lockdowns was deeply concerning. Birdy, 73, described facing the possibility of medical quarantine:

We knew that quarantining was a possibility, but it’s really difficult when you’re put in quarantine. You have no contact with others and no phone. You worry, what’s your family going to think? You don’t have a moment to call your family and say, “hey, look, here’s what’s happening”. If your family tries to call up to the prison system and say “I haven’t heard from [name] in three days, what’s happening?” Most of the time the prison system will not give your family any information […] I would always worry, and I know others worried, about what’s my family going to think if I go into some black hole for two weeks.

Early in the pandemic, the people we spoke with and others inside were reluctant to report symptoms for fear of being isolated. Freedom, 63, described being locked down in her room for 38 days:

Yes. 38 days. The building that I was in, it was like 85% of the ladies there tested positive. It was like 35 of us left, all 35 of us was negative. But during the time they locked us in for 38 days. All we had to do was come out and shower. We had 15 minutes out. During the 15 minutes we got to shower, use the phone or get on our kiosk machines and back in. 15 minutes every day. Yes. For 38 days. And I was very miserable.

The length and specifications of the lockdowns varied across both time and institution. While Freedom described being let out of her room for 15 minutes per day, another woman we spoke with said she was let out only every other day to take a shower. Some described being allowed to go to meals at first, but later having their meals delivered. Cece recalled a period of quarantine after an exposure where she was locked in her cell for ten days. That meant no access to showers or phone, “Nothing,” she said, “10 days, you’re not coming out.”

The effects of the lockdowns were magnified by one’s living environment. Some reported being in dorm-like settings, where they would share a room with 6–8 other people. Others described being locked down in two-person cells. Granny, age 76, offered a description of the space, saying:

And it is very small, you know. If you say how big the room was, I would say it’s probably 10 × 8. But then in that space you have bunk beds that are the size of a regular twin bed so that takes a lot of the space. And then you have a desk. You’ve got two lockers, you’ve got a toilet, you’ve got a sink. So walking space, you might have like 3 feet, if that. So it is very tight.

Being locked down for 23 hours per day or more in such tight quarters had profound effects on the physical and mental health of the women we interviewed. Content described the extreme shift in activity level noting:

I was accustomed to working out and walking […] to suddenly not being able to do anything because they started to lock us in, lock us down. And so there was no movement […] I was gaining weight just sitting on the bed because we couldn’t go anywhere.

Respondents described a clear link between the isolation, lack of control over their physical health and their mental health. Content explained:

So the stress got worse and worse for me to the point that I was crying, I was upset, [because] I couldn’t get out of the room. And it was problematic for me because I have high blood pressure. And so my blood pressure spiked up to almost numbers where they had to start taking my blood pressure every day because they were in the numbers of where I could’ve had a stroke […] It just started to affect my health and mentally it was affecting me. I just felt kind of like helpless.

She went on to describe this period as the most challenging time she had during her entire period of incarceration. Freedom, who also had high blood pressure, described a linkage between her physical and mental health, noting that as her blood pressure rose, so did her anxiety levels. Joy was similarly affected by the extended time in a small space describing, “my anxiety went through the roof […] We had no recourse from it. We just had to sit there and think about it all day long.”

One health-care provider, a nurse, expressed concerns about the physical health effects of lockdowns. She described trying to approach primary care during this time, saying, “there was just no way for people to exercise or move around. And it’s like, here you are telling them to try to make these lifestyle changes and it’s impossible.” The standard recommendations to manage these conditions were completely unavailable to her patients. Granny, who had been very active prior to the pandemic, reflected on the toll of not having any physical activity, saying:

When you’re active and all that activity is taken away from you, that is really another emotional thing. So, I would say the mental health is really deteriorating [for] a lot of people […] You *need* that freedom of movement […] Some people literally cannot stand it and they almost feel like my bunkie. She was saying, “if I can’t get out of here for a while I’m going to kill myself. I can’t take this. I need to do something so I can get out”. And I’m sure she’s not the only one that felt that way.

Other health-care providers expressed similar concerns around the effects of the lockdowns. A nurse who worked part time in a prison reflected on the difference between shelter-in-place policies experienced outside of prisons, and the lockdowns experienced by people who were incarcerated:

It’s different when you’re in prison, right? […] Like you go home, you can go outside in your backyard, you can go outside with masks on to go to the grocery store. Like there’s no *lockdown* lockdown. Like these folks were locked down like 23 hours out of the day. Or even more than that.

Prison, by design, strips away freedoms. Yet, the risk mitigation strategies deployed during the pandemic took away even more.

### Social isolation: “I think the worst thing they can do is what they’ve done: just isolate everybody”

The limited time outside of one’s room also impacted incarcerated people’s ability to maintain meaningful social connections and relationships, including both those inside and outside of prison. As Birdy described, “Prison life is very hard and it’s made harder if you’re isolated from the little support that you get from each other.” This was echoed by Content, who noted, “The little friends that I did have that now is gone because I’m locked in this two-man cell.” While incarcerated, women form deep relationships and care for each other when ill, sad or otherwise in need of support. These relationships may extend across rooms and units and were deeply disrupted by the lockdowns.

The first outside connection that was severed was visitation. Beyond Blessed expressed feeling like the prison was taking one of the few lifelines incarcerated people have to their families on the outside: “March 1 hit, and they took all the visits. No more visits. And it was hard for a lot of people […] there’s no church, there is no outside. It’s just within the unit. So now everybody has got a heightened anxiety, stress, everything.” The lack of visits was devastating for many inside who relied on the visits to maintain relationships with their family, including children and grandchildren. Yet, this was not the only form of connection that was affected by the pandemic. For a time, there was concern about phone calls as a vector of disease spread. Then, even when calls were more readily allowed, the limited time out of one’s cell made it difficult to schedule calls with loved ones. As Content described:

Everything had to be done within that window. To take a shower […] get the cleaning supplies and clean your room, make your phone calls. And there would be times that they would pop the door too late so I could not make a phone call. So that was stressful […]. And it became more and more isolating.

The other primary mode of communication to loved ones outside is a secure email system in which incarcerated individuals can pay to send and receive messages from the outside, which are closely monitored by the prison. Emails are sent through kiosks set up in each unit or tablets available for purchase, which can be used in one’s room to type out emails but must then be synced to the kiosk to send and receive new messages. Those who did not have tablets would need to sit at the kiosks to read, type and send messages. The limits on out-of-cell time meant that many were not always able to use the kiosks as they had in the past. Granny described that individuals were unable to sync their tablets to upload emails. She noted, “nobody had contact with their families. They had to switch over to writing letters again.”

In the absence of other forms of support, many turned to their roommates as the primary source of support during the traumatic events of the pandemic. Granny described this in detail, telling the story of the support she offered to her roommate, who was expressing suicidal ideations:

Oh, I just tell her, “just talk to me […] just tell me what you’re feeling and we’ll talk through it”. […] She had a very rough life, all her life. And you know we talked about her goals. And while I was there I just kept her talking. And she got pretty good to where she could deal with it. And any time something bothered her she said, “have you got a minute?” What else could I have? I’m locked in.

According to Granny, her roommate was able to depend on her as a source of support, but this was not universally available. As she acknowledged, “not everybody wants to listen to somebody else’s problems” nor should they be expected to serve in that role. Further, being locked in with a roommate can also in and of itself be a source of stress. Granny went on to speak of this possibility saying, “And then try to picture being in a room with a roommate who you don’t get along with […] you’re not working so you can’t get away from that person. I mean there’s a lot of tension in the rooms.”

Health-care providers expressed similar concerns about mental health. One physician spoke of her concerns about the lockdowns noting:

I think there is going to be some serious trauma. they are being locked down and have no control over what their exposures are. They have no control over it. They are completely trapped. And I think that is going to create a lot of trauma.

Beyond the pandemic itself as a source of stress and trauma, there was concern expressed about what would happen to those with mental illness subject to lockdowns and quarantined. As a nurse described, “I mean if they have a diagnosis like, you know, severe depression and then you’re locked in a dark room for two weeks is not ideal.” Granny, like many other participants, expressed great concern about the health and well-being of those still inside. In describing the toll lockdowns take on mental health, she noted, “they really need some help. They need to talk to somebody […] other than their roommate. They need to be able to get out and express their feelings without fear.” Unfortunately, for many people inside, there were few options to speak with someone else about their feelings.

### Access to mental health care: “Even if we asked, there was no way for us to get help for our mental disorders”

Mental health structures and services varied by institution. A psychologist we spoke with described the multiple levels of mental health care offered in the women’s prison where she works:

Well, at my prison there are basically three levels of care and then general population, which has no interaction with the mental health system except that they can put in what are called co-pays. So, if they need to see somebody, they’ll get seen. But they’re not like part of the system.

She described a set of ten diagnoses that qualifies someone for care as part of one of the structured programs, but conditions like anxiety do not qualify someone. Another provider described care at his institution similarly, noting that for most of the general population “There’s not one-on-one talk therapy. There’s not like sort of structured treatment.”

Prior to the pandemic, most of the general population who requested mental health care received services via group therapy programs. Many participants we spoke with expressed that they found these programs useful, with one participant, Ms Legacy, 70, describing how many of her friends and family on the outside “need to get in some groups.” Granny spoke of her experience with groups, recalling:

If I wanted to talk, that’s where I’d talk to get it out. And you know, you learn after a lot of years, the only way that you could really get things out and begin to heal is you have to talk about it and let it out. You can’t hold it in. And women are being forced to hold these emotions in.

In the absence of groups, it was difficult to get care or to speak to someone about mental health concerns. As Granny described, “for those of us who are technically normal, it’s very hard to get in there and just talk to somebody. You have to be referred by your staff on the unit. And so I know a lot of people you know, they just lie. They’ll say I want to kill myself or I am hearing voices or you know. Mental help should be available to absolutely everybody whenever in need. And it isn’t.” Granny’s comments convey the desperation some felt to speak to a mental health-care provider, even to the point of inflating their symptoms.

With individuals locked in their cells 23 or more hours per day, the only way to provide non-emergent care for mental health was to talk through the cell door. As Content described:

They would send someone and they would go door-to-door and they would ask us these generic questions. “Do you feel like you want to harm yourself or hurt yourself?” You know, the generic questions. So that was the checking in on us, mentally. And then they would give us papers that we could color or do puzzles and stuff like that in our rooms. It was to me that that was the part they could’ve improved on as far as mental health. Because some of the ladies, they wanted to talk outside […] You know you have to talk through the door, right? Because the door is locked. If you really, really had something going on mentally with you and you really needed to sit down and talk, that was lacking where we didn’t have that outlet to talk to a professional. Because we are not going to yell out through the door what’s really going on with us for everybody to hear. So that was a challenge. So I would say, I can speak for myself, that a lot of my mental issues and things that I was going through and I was trying to work through, I had to work through it on my own based on my self-help groups I’ve taken because I absolutely refused to talk outside the door, yelling so they can hear me what I am going through and how being locked in was affecting me.

This system did not offer any privacy and, in the estimation of those who were incarcerated, made it nearly impossible for a health provider to fully assess someone’s mental state and offer any kind of therapeutic intervention. As described above, CDCR reported a drop in mental health referrals during the pandemic, which fails to reflect the increase in mental health challenges described by both incarcerated individuals and health-care providers working in prisons and jails. As one psychiatrist described it, despite the drop in the incarcerated population that occurred during the pandemic, he felt that “the demand [for mental health services] has never been higher.” He went on to articulate this in more depth:

I just think a lot of people have been not doing great, you know? Not that people were doing great in the jail necessarily before, but a lot of people who I think have just been suffering, they’ve been bored, they’ve been kind of like under a lot of stress. I’ve seen people have a lot of anxiety and I think worse moods, depression issues […] We see people periodically just appear to like spontaneously develop psychosis after a long period of time not being psychotic.

Jail and prison mental health-care providers reported facing structural barriers that challenged their ability to provide care for the general population during the pandemic. Limited staffing and social distancing requirements made it difficult to meet the needs of the general population. Yet, incarcerated people were facing new and worsening stressors with fewer resources to address them. Many participants we interviewed knew someone inside who died by suicide during this period. This was relayed as both an example of a failure of the system – a loved one who did not get the care she needed after displaying signs that her mental health was deteriorating – and also a source of trauma for many inside. One person described someone on her unit who died by suicide, saying:

She had all the signs that she needed mental health care. And staff didn’t put in for observation to have her go talk to mental health […] I see that the system, especially with COVID, they need a lot of resources and they need a lot more personable connection with inmates that need mental health care where they are not talking through the door. I don’t know if they don’t have enough staff, but that is something that needs to be really addressed is mental health care. For all people.

## Discussion

The COVID-19 pandemic – and the risk mitigation strategies implemented in attempt to slow the spread of the disease in carceral settings – have perpetuated a crisis already brewing in US prisons. Although none of our participants was diagnosed with COVID-19 while incarcerated (one reported that they experienced severe illness prior to the advent of COVID-19 testing), our study highlights how carceral COVID-19 risk mitigation strategies intensified the chronic stressors associated with incarceration (Massoglia and Remster, 2019; Porter, 2019) and reinforced a psychological toll on the body. Aging well requires having social support, including strong, connected and safe communities (Radford and Garvey, 2021), and COVID-19 disrupted the limited psychosocial resources incarcerated women depend on to feel connected with their communities both inside and outside of prison. The profound impact of social isolation due to the pandemic has already been demonstrated among older adults (Aronson, 2020) and is magnified in prisons where contact with loved ones both inside and outside the institution has been largely restricted since March 2020.

It is worth noting that the social isolation that occurred as a result of the lockdown and quarantine procedures implemented by CDCR differed in substantial ways from solitary confinement, including being isolated with a roommate and, while at times limited, having access to forms of entertainment and communication (Cloud *et al*., 2020). These differences are meaningful, and we anticipate that the long-term effects of these lockdowns will not mirror the extreme long-term effects of solitary confinement (Haney, 2018). However, the public health benefits of lockdowns must be weighed with the harms, particularly in carceral settings where individuals are already under duress (Porter, 2019) and may nonetheless associate lockdowns with punishment.

While access to COVID-19 vaccinations should ideally lessen the need for intense lockdown procedures, they have continued to be used in institutions with high rates of vaccination. It is important to emphasize that the vast majority of COVID-19 cases within CDCR occurred after lockdown procedures were routinely used; between September 2021 and February 2022, there were more than 20,000 new cases of COVID-19, despite 80% of incarcerated people being fully vaccinated (Population COVID-19 Tracking, 2022). With the rise of new COVID-19 variants, the trajectory of the pandemic remains unclear, yet one thing is certain: the lockdown strategy has not stopped the spread of COVID-19.

Without intervention, COVID-19 mitigation strategies may result in more incarcerated people developing anxiety, depression and other mental health conditions, or the exacerbation of these conditions among those with prior mental health diagnoses. There may be unique consequences for older populations and people of color. While the California State Prison population decreased overall during the COVID-19 pandemic, the proportion of 50 to 69 year-olds increased by 2% and the proportion of individuals who are Black or Hispanic increased by 1% (CDCR, 2021). California did engage in decarceration efforts in 2020, with the aim of easing overcrowding. However, the majority of individuals released through these efforts were those with determinant sentences who were due to be released within 180 days; older adults and/or those with longer sentences were a disproportionately low share of those released (Lyons, 2020).

CDCR has reported that demand for mental health services decreased during the COVID-19 pandemic (Mehta *et al*., 2021). While this may reflect the number of people referred for mental health services during this period, our findings, though focused on older incarcerated women, challenge the assumption that lower demand is indicative of less need for services. Our research indicates that there are structural barriers inside prisons, including lack of staffing, preventing mental health-care workers from providing needed care. CDCR has provided packet-based in-cell activities, and there have been calls to increase the availability of in-cell self-help materials and exercises to combat limited programming and physical movement during the pandemic (Kothari *et al*., 2020). We suggest going further, increasing resources to allow for more one-on-one therapy for those who request it and decreasing the number of hours spent in a cell each day. While we understand the rationale for lockdowns and halting in-person group therapy early in the pandemic, alternative solutions are needed at this phase. Across health care, there has been creative re-imaging of what is possible to meet the needs of vulnerable communities during this challenging time. Carceral institutions could similarly explore ways to use social distancing, masking, outdoor spaces and technology to bring people together for both therapeutic and social purposes. However, given the nation-wide correctional staffing shortage (LeMasters *et al*., 2022), the structural issues our findings illuminate in the delivery of care may persist.

The most straightforward solution to address both the amount of time incarcerated individuals at risk for COVID-19 must spend in their cells and staffing shortages that have limited individual therapy in the general population is decarceration. Both providers we interviewed and medical and public health experts made fervent calls for decarceration early in the pandemic to prevent the large-scale outbreaks that took place over the past two years (Macmadu *et al*., 2020; Oladeru *et al*., 2020; American Public Health Association, 2020). Our research demonstrates that vaccinations have not lessened the need for this type of intervention, and that the effects of the pandemic extend far beyond the morbidity and mortality associated with COVID-19. Decreasing the incarcerated population even further would allow for more social distancing without the need for extreme lockdowns and could allow for resources to be reallocated to meet the needs of those suffering during this stressful time.

COVID-19 has both laid bare and exacerbated the mental health challenges faced by people incarcerated in the USA. It is clear that prisons were designed for punishment, not public health. Our findings raise the question of how we go beyond returning to a pre-pandemic baseline to facilitate community healing on a deeper level. While we must act to address and improve mental health inside prisons, equally important is the need to acknowledge the collective grief that incarcerated people have experienced as a result of lost connections to their families on the outside and communities on the inside, delayed parole and court hearings and deaths of other incarcerated people to COVID-19, suicide and other causes. It is now well understood that COVID-19 inequities have been concentrated in structurally vulnerable environments (Berkowitz *et al*., 2020), disproportionality impacting people of color. As we move toward community-driven, public health solutions, prisons, jails and immigration detention centers must be considered in this conversation. With the most vulnerable patients still incarcerated, the need for physical and mental health-care services will continue to grow.

## Figures and Tables

**Table 1:** Demographic information

*Formerly incarcerated women:*		
*Age*	*N*	%
40–49	3	30
50–59	2	20
60–69	2	20
70+	3	30
Total	10	100
*Race*		
Black	3	30
Black and Latinx	2	20
Pacific Islander	2	20
White	3	30
Total	10	100
*Gender*		
Cisgender woman	7	70
Transgender woman	3	30
Total	10	100
*Years incarcerated*	Range	Median
Range	2–34 years	22 years
Release date	April 2020-December 2020	
*Correctional health-care workers*:		
*Provider type*	*N*	%
Clinical psychologist	1	10
Nurse	4	40
Physician/internist	4	40
Psychiatrist	1	10
Total	10	100
*Race*		
Black	1	10
Pacific Islander	1	10
White	8	80
Total	10	100
*Gender*		
Woman	8	80
Man	2	20
Total	10	100
*Years worked in corrections prior to the pandemic*	Range 0–16 years	
